# Recurrent Pleural and Pericardial Effusions Due to Sarcoidosis

**DOI:** 10.1371/journal.pmed.0020063

**Published:** 2005-03-29

**Authors:** Sankar D Navaneethan, Sundar Venkatesh, Rakesh Shrivastava, Jagat Mehta, Robert Israel

**Affiliations:** Department of Internal Medicine, Unity Health System, Rochester, New YorkUnited States of America

## Abstract

A 54-year-old man presented with fever, shortness of breath, and left-sided pleuritic chest pain. His bilateral pleural effusions and pericardial effusion turned out to be due to sarcoidosis

## PRESENTATION of CASE

A 54-y-old white male presented to the hospital during the winter of 2002 with complaints of fever, shortness of breath, and left-sided pleuritic chest pain of 2 d duration. He was a nonsmoker without any significant family history of pulmonary disease. He was retired, and denied any past exposure to chemicals including beryllium. On physical examination, the patient was febrile with tachycardia and normal blood pressure. Air entry at both lung bases was diminished. The remainder of his history and physical examination was unremarkable.

He had been hospitalized twice before for similar complaints during the prior 6 wk. Investigations during the first admission showed mild leukocytosis with normal electrolytes. Chest radiograph at that time showed bilateral pleural effusions and cardiomegaly, while echocardiography showed cardiac tamponade for which he underwent emergency pericardiocentesis. A computed tomography (CT) scan of the chest after the pericardiocentesis showed bilateral pleural effusions without hilar or mediastinal lymphadenopathy. A CT scan of the abdomen and pelvis, to look for any occult malignancy, was normal. Therapeutic thoracentesis was performed to relieve his symptoms. Pleural and pericardial fluid analyses were exudative without malignant cells, and were negative on culture for bacterial, mycobacterial, and fungal organisms. Investigations for HIV, syphilis, rheumatological diseases, hepatitis, and occult malignancies were all negative. The patient improved symptomatically with pericardiocentesis and thoracentesis, and he was discharged with the differential diagnosis of an atypical vasculitic syndrome or a paraneoplastic syndrome.

On the second admission, he presented with increasing shortness of breath, and a CT scan of the chest showed recurrent bilateral pleural effusions without pulmonary disease. Therapeutic thoracentesis was performed, relieving his symptoms, and he was discharged.

**Figure 1:**
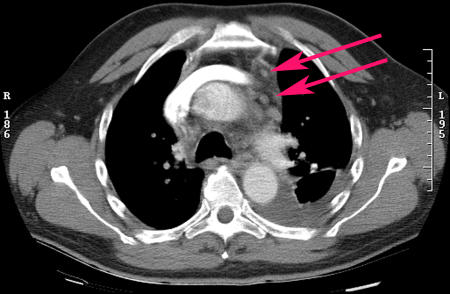
CT of the Chest during the Third Hospitalization The CT shows mediastinal lymphadenopathy (pink arrows) and left pleural effusion.

During his third (current) admission, laboratory investigations were unremarkable. Repeat CT scan of the chest showed a left-sided pleural effusion, normal lung parenchyma, a small pericardial effusion, and mediastinal lymph nodes (Figure 1). Video-assisted thoracoscopic pleural biopsy showed nonspecific chronic inflammation. Mediastinal lymph node biopsy showed benign reactive lymph nodes with focal epithelioid, non-caseating granulomas consistent with sarcoidosis. The patient was treated with oral prednisone, 20 mg daily. He remained asymptomatic when seen at his 1-y follow-up, when he was taking 7.5 mg of prednisone daily.

## DISCUSSION

Sarcoidosis is characterized by non-caseating granulomas in affected organs. It primarily affects the lungs. Pleural effusion occurs in 5% of patients and may be the presenting feature of the disease [1]. Cardiac manifestations include bundle branch block, arrhythmia, congestive heart failure, pericarditis, and cardiomyopathy. Asymptomatic minimal pericardial effusion has been shown to occur in 20% of cases [2,3]. Sarcoidosis presenting with pleural and pericardial effusion is extremely rare, and only one previous case has been reported [4].

Patients presenting with coincident pleural and pericardial effusions need to be investigated for rheumatological diseases, occult malignancies, and chronic infections such as HIV, tuberculosis, hepatitis, and syphilis. The diagnosis of sarcoidosis requires the presence of clinical and radiographic findings suggestive of sarcoidosis, non-caseating granulomas found on biopsies obtained from one or more sites, and the exclusion of other granuloma-forming diseases [5]. The differential diagnosis of non-caseating granulomas includes mycobacterial infections, berylliosis, and sarcoidosis. Our patient had clinical and radiological features suggestive of sarcoidosis and had non-caseating granulomas on his mediastinal lymph node biopsy, and we excluded other possible causes of recurrent pleural and pericardial effusions.

The optimal management strategy for sarcoidosis still remains uncertain. Asymptomatic pulmonary sarcoidosis is best treated with a wait-and-watch approach [6]. But steroids remain the mainstay of treatment for systemic sarcoidosis involving the cardiovascular system, nervous system, or eyes, and for cases with progressive pulmonary involvement [7,8]. Earlier institution of steroids in cardiac sarcoidosis may prevent progressive disease and improve outcomes [9,10]. Cytotoxic agents are used as steroid-sparing agents in patients requiring large doses of steroids and who experience serious side effects [11].

**Learning Points**

• Patients with recurrent pleural and pericardial effusion should be investigated for chronic infections, rheumatological illnesses, and malignancies.

• Sarcoidosis should be included in the differential diagnosis of patients presenting with bilateral pleural and pericardial effusion, as early treatment may improve the outcome in cardiac sarcoidosis.

• Steroids remain the mainstay of treatment for systemic sarcoidosis involving the cardiovascular system, nervous system, or eyes, and for cases with progressive pulmonary involvement.

## Abbreviation

CT: computed tomography

## References

[pmed-0020063-b01] Salazar A, Mana J, Corbella X, Vidaller A (1994). Sarcoid pleural effusion: A report of two cases. Sarcoidosis.

[pmed-0020063-b02] Angomachalelis N, Hourzamanis A, Salem N, Vakalis D, Serasli E (1994). Pericardial effusion concomitant with specific heart muscle disease in systemic sarcoidosis. Postgrad Med J.

[pmed-0020063-b03] Israel RH, Poe RH (1994). Massive pericardial effusion in sarcoidosis. Respiration.

[pmed-0020063-b04] Krawczyk I, Sedlaczek AM (1997). A case of sarcoidosis with massive pleural and pericardial effusion. Pneumonol Alergol Pol.

[pmed-0020063-b05] (1999). Statement on sarcoidosis. Joint Statement of the American Thoracic Society (ATS), the European Respiratory Society (ERS) and the World Association of Sarcoidosis and Other Granulomatous Disorders (WASOG) adopted by the ATS Board of Directors and by the ERS Executive Committee, February 1999. Am J Respir Crit Care Med.

[pmed-0020063-b06] Gibson GJ, Prescott RJ, Muers MF, Middleton WG, Mitchell DN (1996). British Thoracic Society Sarcoidosis study: Effects of long term corticosteroid treatment. Thorax.

[pmed-0020063-b07] Paramothayan NS, Jones PW (2000). Corticosteroids for pulmonary sarcoidosis. Cochrane Database Syst Rev.

[pmed-0020063-b08] Newman LS, Rose CS, Maier LA (1997). Sarcoidosis. N Engl J Med.

[pmed-0020063-b09] Skiguchi M, Yazaki Y, Isobe M, Hiroe M (1996). Cardiac sarcoidosis: Diagnostic, prognostic, and therapeutic considerations. Cardiovasc Drugs Ther.

[pmed-0020063-b10] Syed J, Myers R (2004). Sarcoid heart disease. Can J Cardiol.

[pmed-0020063-b11] Paramothayan S, Lasserson TJ, Walters EH (2003). Immunosuppressive and cytotoxic therapy for pulmonary sarcoidosis. Cochrane Database Syst Rev.

